# Clozapine Prescribing in the General Hospitals

**DOI:** 10.1192/bjo.2025.10527

**Published:** 2025-06-20

**Authors:** Hanna Tee, Namrata Soni, Kara Hosegood, Dhaveena Rajathevan

**Affiliations:** 1 Dorset Healthcare University NHS Foundation Trust, Bournemouth, United Kingdom; 2 University Hospitals Dorset NHS Foundation Trust, Bournemouth, United Kingdom

## Abstract

**Aims:** Clozapine is an effective treatment for adults with schizophrenia, who have not responded to two other antipsychotic medications. However, there are challenges prescribing when patients who take clozapine in the community are admitted to a General Hospital for physical health reasons. These challenges can include physical health co-morbidities, medication interactions, blood dyscrasias, changes in smoking status and bowel movements, infection, missed or incorrect doses of clozapine prescribed, and clozapine toxicity.

Our aim was to evaluate the management of clozapine for patients admitted to a General Hospital, including the interaction with Liaison Psychiatry.

**Methods:** Data were collected retrospectively, between January 2022 and January 2023, from two General Hospitals in England. There were a total of 45 admissions to a General Hospital for 34 patients who were identified as prescribed clozapine in the community. Electronic records were accessed for the community, General Hospital and Mental Health service. Records were reviewed to assess if the clozapine prescription was correctly recorded on the community record, and if a referral to Liaison Psychiatry was made on admission. Within the Liaison Psychiatry review, records were reviewed for documentation of full blood count, medication concordance, smoking status, bowel movements, physical health concerns, medication interactions, signs of clozapine toxicity and recommendations for a clozapine level.

**Results:** The clozapine prescription was documented correctly in 16% (7/45) of occurrences in community records.

On admission to a General Hospital, 49% (22/45) of patients were referred to Liaison Psychiatry. The mean time for referral from admission was 41.07 hours. Of the 22 admissions that were referred, 68% (15/22) were seen within 24 hours by Liaison Psychiatry.

On review with Liaison Psychiatry, the frequency of documentation seen was: full blood count (65%), medication concordance (65%), smoking status (50%), bowel movements (41%), physical health concerns (91%), medication interactions (38%) and signs of clozapine toxicity (18%). Advice regarding a clozapine level was documented for 35% of patients.

**Conclusion:** Local education was arranged for the community, General Hospital and Mental Health Trust. A Trust-wide policy was written for the General Hospital to utilise for patients that are prescribed clozapine. This included the importance of referring immediately to Liaison Psychiatry to reduce disruption in treatment and the need to monitor for bowel-related complications. A clozapine admission checklist was introduced for local Psychiatry teams to use when reviewing a patient who takes clozapine. With these measures now implemented, data will be collected to review the effectiveness.

